# An economic and ecological perspective of ethanol production from renewable agro waste: a review

**DOI:** 10.1186/2191-0855-2-65

**Published:** 2012-12-07

**Authors:** Latika Bhatia, Sonia Johri, Rumana Ahmad

**Affiliations:** 1 Department of Life Sciences, Institute of Technology & Management University, Gwalior, Madhya Pradesh, 475001, India

**Keywords:** Bioethanol, Lignocellulosic waste, Immobilization, Mutagenesis, SSF

## Abstract

Agro-industrial wastes are generated during the industrial processing of agricultural products. These wastes are generated in large amounts throughout the year, and are the most abundant renewable resources on earth. Due to the large availability and composition rich in compounds that could be used in other processes, there is a great interest on the reuse of these wastes, both from economical and environmental view points. The economic aspect is based on the fact that such wastes may be used as low-cost raw materials for the production of other value-added compounds, with the expectancy of reducing the production costs. The environmental concern is because most of the agro-industrial wastes contain phenolic compounds and/or other compounds of toxic potential; which may cause deterioration of the environment when the waste is discharged to the nature. Although the production of bioethanol offers many benefits, more research is needed in the aspects like feedstock preparation, fermentation technology modification, etc., to make bioethanol more economically viable.

## Introduction

Since the late 19^th^ century, the mean temperature on earth has increased with 0.8°C and the major part of this increase is likely due to anthropogenic emissions of greenhouse gases. Carbon dioxide is the type of greenhouse gas with largest emission and this originates from the combustion of fossil fuels as coal, oil and natural gas (Sun and Cheng 2002). In USA, transportation accounts for 30% of the total energy consumption. Burning fossil fuels such as coal and oil releases CO_2_, which is a major cause of global warming. With only 4.5% of the world’s population, the US is responsible for about 25% of global energy consumption and 25% of global CO_2_ emissions. The average price of gasoline in 2005 was $2.56 per gallon, which was $0.67 higher than the average price of gasoline in the previous year. Yet in June 2008, the average price of gasoline in the US reached $4.10 per gallon (Kumar et al. 2009).

Soaring oil prices associated with concerns of climate change and national energy security are driving us to utilize sustainable alternative energy sources, such as solar energy, nuclear energy, wind energy, hydropower, tidal energy, and so on (Lynd et al. 2003). With the inevitable depletion of world’s energy supply, there has been an increasing interest worldwide in alternative sources of energy. Unlike fossil fuels, ethanol is a renewable energy source produced through fermentation of sugars and used as a partial gasoline replacement in a few countries of the world (Sharma et al. 2007).

Bioethanol market is expected to reach 100 x10^9^ liters in 2015. The largest producers in the world are the US, Brazil, and China. In 2009, US produced 39.5x10^9^ liters of ethanol using corn as a feedstock while the second largest producer, Brazil, created about 30x10^9^ liters of ethanol using sugarcane. China is a country that has invested much in the production of ethanol, and is nowadays one of the largest ethanol producers (Ivanova et al. 2011).

Ethanol contains 35% oxygen, which results in a complete combustion of fuel and thus lowers the emission of harmful gases. Moreover, ethanol production uses energy from renewable sources only; hence, no net carbon dioxide is added to the environment, thus reducing green-house gas emissions. It has also been well established now that ethanol increases the octane number, decreases the Reid vapor pressure and produces fuel with clean burning characteristics (Dhillon et al. 2007). Moreover, neat (unblended) ethanol can be burned with greater efficiency, and is thought to produce smaller amounts of ozone precursors (thus decreasing urban air pollution), and is particularly beneficial with respect to low net CO_2_ put into the atmosphere.

The increasing demand for various industrial purposes such as alternative source of energy, industrial solvents, cleansing agents and preservatives, has necessitated increased production of ethanol (Brooks 2008). Furthermore, ethanol by fermentation offers a more favorable trade balance, enhanced energy security, and a major new crop for a depressed agricultural economy. Ethanol is considerably less toxic to humans than is gasoline (or methanol). Ethanol also reduces smog formation because of low volatility; its photochemical reactivity and low production of combustion products. Furthermore, low levels of smog-producing compounds are formed by its combustion (Wyman and Hinman 1990). In addition, the low flame temperature of ethanol results in good engine performance.

**Figure 1:**
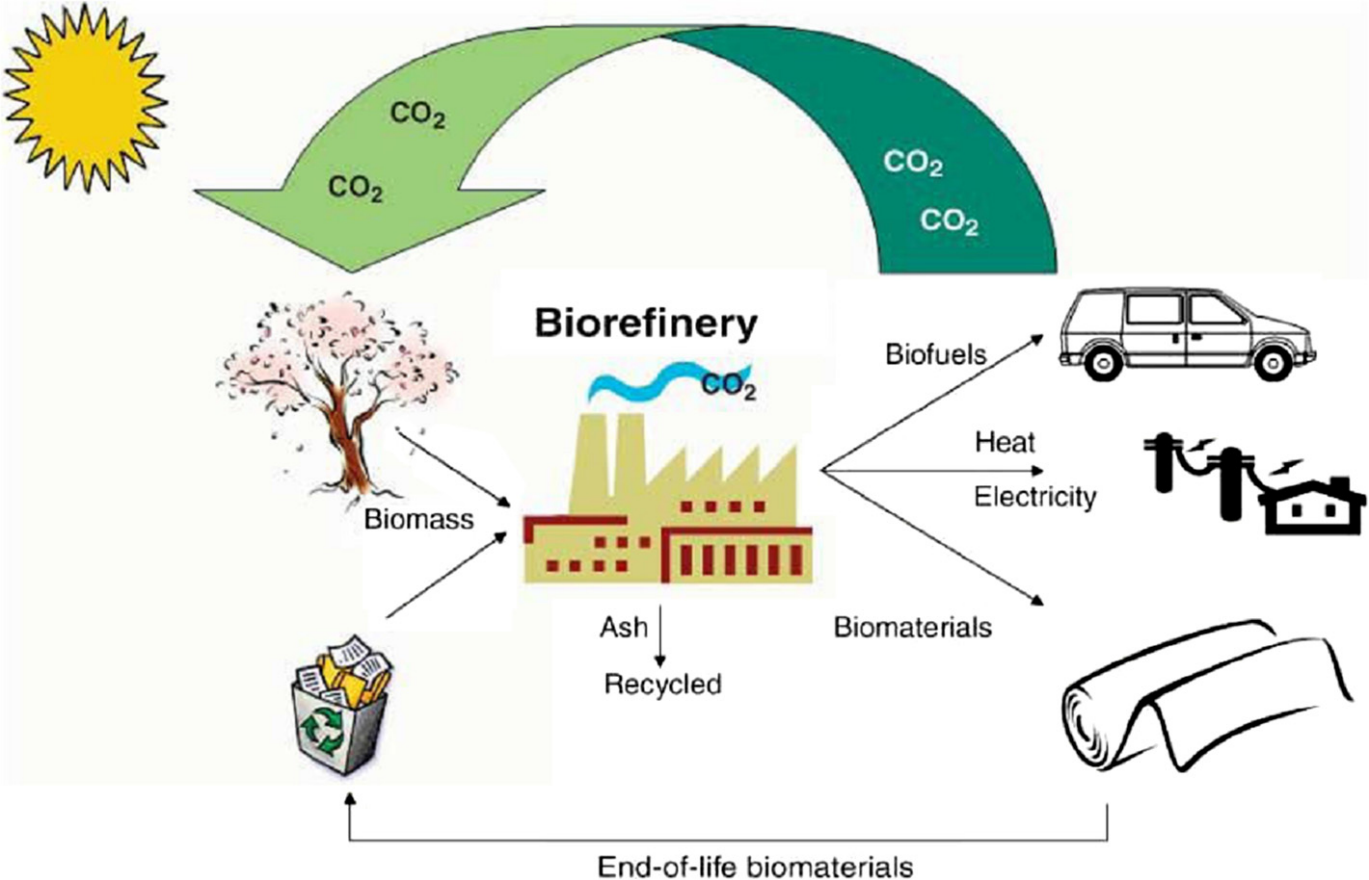
**The fully integrated agro-biofuel-biomaterial-biopower cycle for sustainable technologies.** (Ragauskas et. al Ragauskas et al. 2006).

Currently, bioethanol is being commercially produced only from edible feedstock such as corn-starch and sugarcane juice. The European Union (EU) had established a goal of 5.75% biomass-derived transportation fuels by December, 2010. The use of fuel ethanol has been quite successful in Brazil, where it is being produced at a very low cost by fermentation of sugarcane. In the US, corn is the dominant biomass feedstock for production of ethanol, and in the EU, straw and other agricultural wastes are the preferred types of biomass for ethanol production (Raposo et al. 2009). These bioethanol production systems pose a concern about competition with food and feed supplies. To avoid this competition, bioethanol production from non edible lignocellulosic biomass such as wheat straw, rice straw, bagasse, corn stover, wood, peels of fruits and vegetables is attracting keen interest. The current production and use of bioethanol processes are a starting point. It is our belief that the next generational change in the use of bioresources will come from a total integration of innovative plant resources, synthesis of biomaterials, and generation of biofuels and biopower (Figure 1).

The present review is a concise overview of current and latest developments in ethanol production with special emphasis on the choice of lignocellulosic substrates, pretreatment methods and types of microorganisms that have been used for optimal, ecological and economic production of ethanol. Also reviewed are the different fungal and bacterial lignocellulolytic enzymatic systems including the current status of the technology for bioconversion of lignocellulose residues by microorganisms (particularly yeasts and fungi), with focus on the most economical and eco-friendly method for ethanol production.

### Lignocellulosic biomass

Lignocellulose is a renewable organic material and is the major structural component of all plants. Lignocellulose consists of three major components:

i. Cellulose, the major constituent of all plant material and the most abundant organic molecule on earth, is a linear biopolymer of anhydroglucopyranose-molecules, connected by β-1, 4-glycosidic bonds. Cellulose or β-1-4-glucan is a linear polysaccharide polymer of glucose made of cellobiose units. The cellulose chains are packed by hydrogen bonds in so-called ‘elementary microfibrils’. These fibrils are attached to each other by hemicelluloses, amorphous polymers of different sugars as well as other polymers such as pectin, and covered by lignin. The microfibrils are often associated in the form of bundles or macrofibrils. This special and complicated structure makes cellulose resistant to both biological and chemical treatments. (Delmer and Amor 1995, Morohoshi 1991, Ha et al. 1998).

ii. Hemicellulose, the second most abundant component of lignocellulosic biomass, is a heterogeneous polymer of pentoses (including xylose and arabinose), hexoses (mainly mannose, less glucose and galactose) and sugar acids. Hemicellulose is less complex, its concentration in lignocellulosic biomass is 25 to 35% and it is easily hydrolysable to fermentable sugars (Saha et al. 2007). The dominant sugars in hemicelluloses are mannose in softwoods and xylose in hardwoods and agriculture residues (Persson et al. 2006, Lavarack et al. 2002, Balan et al. 2009).

iii. Lignin, the third main heterogeneous polymer in lignocellulosic residues, generally contains three aromatic alcohols including coniferyl alcohol, sinapyl and *p*-coumaryl. Lignin serves as a sort of ‘glue’ giving the biomass fibers its structural strength. Lignin acts as a barrier for any solutions or enzymes by linking to both hemicelluloses and cellulose and prevents penetration of lignocellulolytic enzymes to the interior lignocellulosic structure. Not surprisingly, lignin is the most recalcitrant component of lignocellulosic material to degrade (Zaldivar et al. 2001, Hamelinck et al. 2005).

### Lignocellulose substrates used for ethanol production

Sweet sorghum bagasse can be converted efficiently into fermentable sugars (and is a new potential raw material for fuel ethanol production) by SO_2_ catalyzed steam pretreatment at 190°C for 10 min or 200°C for 5 min followed by enzymatic hydrolysis with a result of 89-92% glucan conversion (Sipos et al. 2009). Hemp and ensiled hemp can be converted into ethanol with steam pretreatment (2% SO_2_ catalyst, 210°C for 5 min) followed by simultaneous saccharification and fermentation at high solid loading (7.5% water insoluble solids[WIS]) with a result of 171–163 g ethanol/kg raw material (Sipos et al. 2010). In Brazil, ethanol is usually produced from cane juice, whereas in USA, starch-crops such as corn are usually used for ethanol production (Sanchez 2009). Using sugars or corn as the main source for ethanol production caused a great deal of controversy due to its effect on food production and costs, which has made it difficult for ethanol to become cost competitive with fossil fuels. These concerns became a driving force in the generation of new biofuel research using lignocellulosic wastes produced by many different industries. Apart from corn and cane juice; wheat, oat and barley straw has also been routinely used to produce up to 0.52 million gallons of ethanol per year (Hahn et al. 2006). China is the world’s largest sweet potato (*Ipomoea batatas* Lam.) producer (accounting for 85% of global production), with the output exceeded 100 M tons in 2005 (Lu et al. 2006). Zhang et al. ( 2011) reported sweet potato as an attractive feedstock for bioethanol production from both economic and environment friendly standpoints.

Lignocellulosic wastes are produced in large amounts by different industries including forestry, pulp and paper, agriculture and food, in addition to different wastes from municipal solid waste (MSW), and animal wastes (Sims 2003, Kim and Dale 2004, Kalogo et al. 2007, Champagne 2007, Wen et al. 2004). Those derived from agricultural activities include materials such as straw, stem, stalk, leaves, husk, shell, peel, lint, seed/stones, pulp or stubble from fruits, legumes or cereals (rice, wheat, corn, sorghum, barley), bagasses generated from sugarcane or sweet sorghum milling, spent coffee grounds, brewer’s spent grains, and many others. These potentially valuable materials were treated as waste in many countries in the past, and still are today in some developing countries, which raises many environmental concerns (Palacios-Orueta et al. 2005). Significant efforts, many of which have been successful, have been made to convert these lignocellulosic residues to valuable products such as biofuels, chemicals and animal feed (Howard et al. 2003). Banana peel, an agro waste can be used as a substrate for ethanol production owing to its rich carbohydrate, crude proteins and reducing sugars. Moreover, banana peels are affordable and renewable low cost raw material which makes it potential feedstock for ethanol production (Bhatia and Paliwal 2010). Similarly pineapple is the second harvest of importance after bananas, contributing to over 20% of the world production of tropical fruits (Coveca 2002). Thailand, Philippines, Brazil and China are the main pineapple producers in the world supplying nearly 50% of the total output. Other important producers include India, Nigeria, Kenya, Indonesia, México and Costa Rica and these countries provide most of the remaining fruit available (50%). Isitua and Ibeh 2010 assayed the feasibility of obtaining ethanol from pineapple waste with the purpose of obtaining a valuable product from the residues of the juice and canning industries.

Large volume of bagasse is generated during sugarcane processing. Agricultural profitability and environmental protection issues are associated with disposal of bagasse. In recent years, potential efforts have been directed towards the utilization of cheap renewable agricultural resources, such as sugarcane bagasse as alternative substrate for ethanol production (Bhatia and Paliwal 2011). Rice is the major crop grown worldwide with an annual productivity around 800 million metric tonnes that corresponds with the large production of rice straw. In search for viable alternatives of biofuels, paddy straw has been pursued as suitable lignocellulosic waste for ethanol production (Wati et al. 2007).

Feasibility of lignocellulosic material for ethanol production has been explored around the world depending upon availability. Production of ethanol from wheat straw, one of the most abundant agricultural wastes, has been extensively studied (Ballesteros et al. 2004, Curreli et al. 2002, Curreli et al. 1997, Talebnia et al. 2010). The average yield of wheat straw is 1.3–1.4 lb per lb of wheat grain (Montane et al. 1998). According to Ballesteros et al. 2006, under the 60% ground cover practice, about 354 millions of tons of wheat straw could be available globally and could produce 104 GL of bioethanol. Europe production would account for about 38% of this world bioethanol capacity. In Spain, grain industry generates important amounts of wheat straw, a part of which is used as bedding straw and the remainder is burned or left on the land to fertilize the soil. Bioconversion of this residue to fuel ethanol would provide an attractive possibility to boost the development of biofuels in a sustainable way.

### Overview of lignocellulosic fermentation

**Figure 2:**
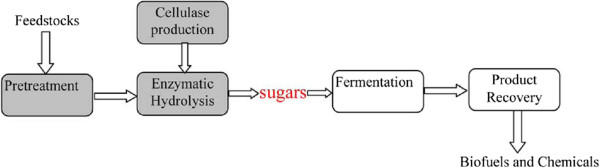
**Major steps involved in the conversion of lignocellulosic biomass to ethanol (Dashtban et al.**^2009^**).**

Schematic picture for the conversion of lignocellulosic biomass to ethanol, including the major steps can been seen in Figure 2. Pretreatment of the lignocellulosic residues is necessary because hydrolysis of non-pretreated materials is slow, and results in low product yield. Some pretreatment methods increase the pore size and reduce the crystallinity of cellulose (Dawson and Boopathy 2007). Pretreatment also makes cellulose more accessible to the cellulolytic enzymes, which in return reduces enzyme requirements and, thus, the cost of ethanol production. The pretreatment not only enhance the bio-digestibility of the wastes for ethanol production, but also results in enrichment of the difficult biodegradable materials, and improves the yield of ethanol from the wastes.

Post pretreatment, the recalcitrant lignocellulosic biomass becomes susceptible to acid and/or enzymatic hydrolysis as the cellulosic microfibrils are exposed and/or accessible to hydrolyzing agents (Jacobsen and Wyman 2000). In the pretreatment process, small amounts of cellulose and most of hemicellulose is hydrolyzed to sugar monomers; mainly D-xylose and D-arabinose. The pretreated biomass is then subjected to filtration to separate liquids (hemicellulose hydrolysate) and solid (lignin and cellulose). After detoxification, the liquid is sent to a xylose (pentose) fermentation column for ethanol production. Solids are subjected to hydrolysis (also called second stage hydrolysis). This process is mainly accomplished by enzymatic methods using cellulases. Mild acid hydrolysis using sulfuric and hydrochloric acids is an alternative procedure (Zhang and Lynd 2004). The hydrolyzed sugars such as D-glucose, D-galactose, and D-mannose, can be readily fermented to ethanol using various strains of *Saccharomyces cerevisae*. The pentoses (D-xylose and D-arabinose) from hemicellulose hydrolysis are not easily utilized by *Saccharomyces* strains; therefore, genetically modified strains of *Pichia stipitis*, *Zymomonas mobilis*, are used for their fermentation. *Candida shehatae* is capable of co-fermenting both pentoses and hexoses to ethanol and other value-added products at high yields (Betancur 2005, Senthilkumar and Gunasekaran 2005).

**Figure 3:**
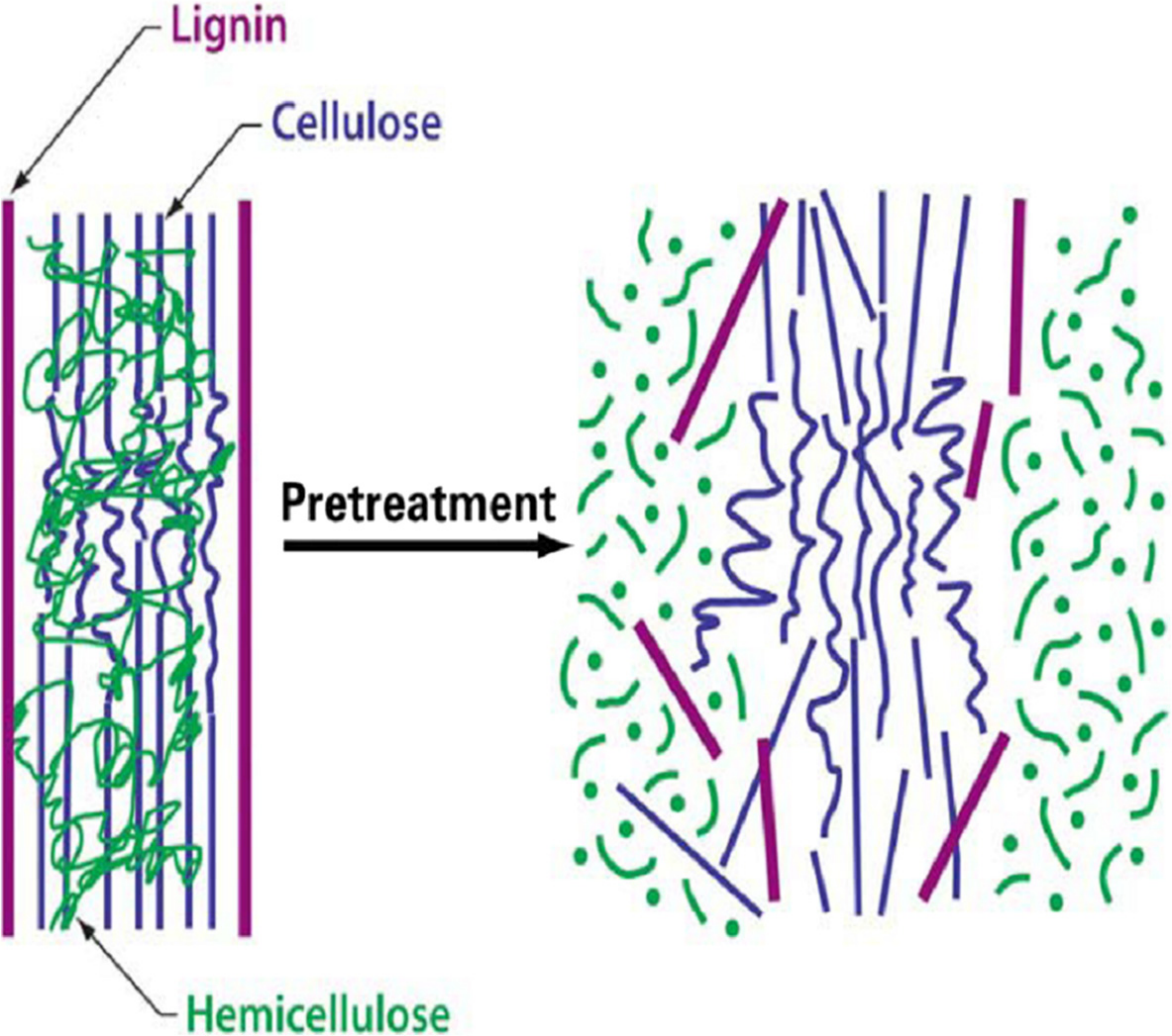
Schematic of goals of pretreatment on lignocellulosic material.

Numerous pretreatment strategies have been developed to enhance the reactivity of cellulose and to increase the yield of fermentable sugars. Typical goals of pretreatment include:

i. Production of highly digestible solids that enhances sugar yields during enzyme hydrolysis, avoidance of degradation of sugars (mainly pentoses) including those derived from hemicelluloses.

ii. Minimization of formation of inhibitors for subsequent fermentation steps.

iii. Recovery of lignin for conversion into valuable co-products.

iv. Cost effectiveness by operating in reactors of moderate size and by minimizing heat and power requirements (Mosier et al. 2005, Sun and Cheng 2007, Yang and Wyman 2008). Figure 3 depicts schematic of goals of pretreatment on lignocellulosic material.

### Physical pretreatments methods

Physical pretreatments methods such as ball milling and grinding have been used for degradation of lignocelluloses with limited success. This method of pretreatment being cost effective and ecofriendly, and one on which relatively little work has been done and reported, so far, would form one of the thrust areas of future research.

Waste materials can be comminuted by a combination of chipping, grinding and milling to reduce cellulose crystallinity. The size of the materials is usually 10–30 mm after chipping and 0.2–2 mm after milling or grinding. Vibratory ball milling has been found to be more effective in breaking down the cellulose crystallinity of spruce and aspen chips and improving the digestibility of the biomass than ordinary ball milling (Millet et al. 1976). The power requirement of mechanical comminution of agricultural materials depends on the final particle size and the waste biomass characteristics (Cadoche and Lopez 1989).

Pyrolysis has also been used for pretreatment of lignocellulosic materials. When the materials are treated at temperatures greater than 300°C, cellulose rapidly decomposes to produce gaseous products and residual char (Kilzer and Broido 1965, Shafizadeh and Bradbury 1979). The decomposition is much slower and less volatile products are formed at lower temperatures.

The efficiency of ultrasound in the processing of vegetal materials has been already proved (Vinatoru et al. 1999). The known ultrasounds benefits, such as swelling of vegetal cells and fragmentation due to the cavitational effect associated with the ultrasonic treatment, act by increasing the yield and by shortening of the extraction time. The effect of ultrasound on lignocellulosic biomass has been employed in order to improve the extractability of hemicelluloses (Ebringerova et al. 2002), cellulose (Pappas et al. 2002), lignin (Sun and Tomkinson 2002) or to get clean cellulosic fiber from used paper (Scott and Gerber 1995) but only few attempts to improve the susceptibility of lignocellulosic materials to biodegradation by using ultrasound have been described. It was found out that ultrasound has a beneficial effect on saccharification processes (Rolz 1986). Sonication has been reported to decrease cellulase requirements by 1/3 to 1/2 and to increase ethanol production from mixed waste office paper by approximately 20% (Wood et al. 1997). It was notice that the effect of ultrasound fragmentation of Avicel (microcrystalline cellulose formed by acid treatment) is similar to that of the enzymes for short incubation intervals (Gama et al. 1997). The time needed for ultrasonic treatment could be reduced when increasing the irradiation power (Imai et al. 2004).

### Chemical pretreatment methods

#### Alkaline pretreatment

Alkaline pretreatment involves the use of bases, such as sodium, potassium, calcium, and ammonium hydroxide, for the pretreatment of lignocellulosic biomass. The use of an alkali causes the degradation of ester and glycosidic side chains resulting in structural alteration of lignin, cellulose swelling, partial decrystallization of cellulose (Cheng et al. 2010, Ibrahim et al. 2011, McIntosh et al. 2010) and partial solvation of hemicelluloses (McIntosh et al. 2010, Sills et al. 2011). Sodium hydroxide has been extensively studied for many years, and it has been shown to disrupt the lignin structure of the biomass, increasing the accessibility of enzymes to cellulose and hemicellulose (MacDonald et al. 1983, Soto et al. 1994, Zhao et al., 2008). Another alkali that has been used for the pretreatment of biomass is lime. Lignocellulosic feedstocks that have been shown to benefit from this method of pretreatment are corn stover, switchgrass, bagasse, wheat, and rice straw.

The conditions for alkaline pretreatment are usually less severe than other pretreatments. It can be performed at ambient conditions, but longer pretreatment times are required than at higher temperatures. The advantage of lime pretreatment is that the cost of lime required for pretreatment of a given quantity of biomass is lowest among alkaline treatments. Most commonly used alkali in the alkali pretreatment processes are NaOH and Ca(OH)_2_. This process results in (i) the removal of all lignin and part of hemicellulose, and (ii) increased reactivity of cellulose in further hydrolysis steps (Hamelinck et al. 2005), especially, enzymatic hydrolysis. Effective removal of lignin minimizes adsorption of enzyme onto lignin and thus allows for effective interactions with cellulose (Aswathy et al. 2010). Between NaOH and Ca(OH)_2_, pretreatment with Ca(OH)_2_ is preferable because it is less expensive, more safer as compared to NaOH and it can be easily recovered from the hydrolysate by reaction with CO_2_ (Mosier et al. 2005).

#### Acid pretreatment methods

Acid pretreatment involves the use of concentrated and diluted acids to break the rigid structure of the lignocellulosic material. The most commonly used acid is dilute sulphuric acid (H_2_SO_4_), which has been commercially used to pre-treat a wide variety of biomass types-switchgrass, corn stover, spruce (softwood), and poplar. Acid pretreatment (removal of hemicellulose) followed by alkali pretreatment (removal of lignin) has shown to yield relatively pure cellulose (Wingren et al. 2003, Taherzadeh and Karimi 2008). Strong acid allows complete breakdown of the components in the biomass to sugars, but also requires large volumes of concentrated sulfuric acid and can result in the production of furfural, an inhibitory byproduct (Goldstein and Easter 1992). Dilute acid allows reduced acid concentrations, but requires higher temperatures, and again gives furfural.

A key advantage of acid pretreatment is that a subsequent enzymatic hydrolysis step is sometimes not required, as the acid itself hydrolyses the biomass to yield fermentable sugars (Zhu et al. 2009). A mixture of H_2_SO_4_ and acetic acid resulted in 90% saccharification (DeMoraes-Racha et al. 2010). Hemicellulose and lignin are solubilized with minimal degradation, and the hemicellulose is converted to sugars with acid pretreatment. The major drawback to these acid processes is the cost of acid and the requirement to neutralize the acid after treatment.

#### Wet oxidation

Wet oxidation utilizes oxygen as an oxidizer for compounds dissolved in water. Typically, the procedure for wet oxidation consists of drying and milling lignocellulosic biomass to obtain particles that are 2 mm in length, to which water is added at a ratio of 1 L to 6 g biomass. Wet oxidation has been used to fractionate lignocellulosic material by solubilizing hemicellulose and removing lignin (Martin et al. 2007, Banerjee et al. 2009). It has been shown to be effective in pretreating a variety of biomass such as wheat straw, corn stover, sugarcane bagasse, cassava, peanuts, rye, canola, faba beans, and reed to obtain glucose and xylose after enzymatic hydrolysis (Martin et al. 2008, Banerjee et al. 2009, Ruffell et al. 2010, Szijarto et al. 2009, Martin and Thomsen 2007). During wet oxidation, lignin is decomposed to carbon dioxide, water and carboxylic acids. Biomass such as straw, reed and other cereal crop residues have a dense wax coating containing silica and protein which is removed by wet oxidation (Schmidt et al. 2002).

Wet oxidation has been combined with other pretreatment methods to further increase the yield of sugars after enzymatic hydrolysis. Combining wet oxidation with alkaline pretreatment has been shown to reduce the formation of byproducts, thereby decreasing inhibition. Bjerre et al. ( 1996) used wet oxidation and alkaline hydrolysis of wheat straw (20 g straw/l, 170°C, 5–10 min), and achieved 85% conversion yield of cellulose to glucose. Wet oxidation combined with base addition readily oxidizes lignin from wheat straw, thus making the polysaccharides more susceptible to enzymatic hydrolysis. Furfural and hydroxymethylfurfural, known inhibitors of microbial growth when other pretreatment **s**ystems are applied, were not observed following the wet oxidation treatment (Azzam 1989).

#### Green solvents

Processing of lignocellulosic biomass with ionic liquids (IL) and other solvents has gained importance in the last decade due to the tunability of the solvent chemistry and hence the ability to dissolve a wide variety of biomass types. Ionic liquid (IL) was found to possess a great potential in dissolving cellulose (Swatloski et al. 2002). Ionic liquids are salts, typically composed of a small anion and a large organic cation, which exist as liquids at room temperature and have very low vapor pressure. The chemistry of the anion and cation has been tuned to generate a wide variety of liquids which can dissolve a number of biomass types-corn stover (Cao et al. 2010), cotton (Zhao et al. 2009), bagasse (Wang et al. 2009), switchgrass, wheat straw (Li et al. 2009).

Dadi and coworkers ( 2007) have studied the enzymatic hydrolysis of Avicel regenerated from two different ILs, 1-*n*-butyl-3-methylimidazolium chloride and 1-allyl-3-methylimidazoliumchloride. Hydrolysis kinetics of the IL-treated cellulose was significantly enhanced. A limitation in using ionic liquids is the fact they tend to inactivate cellulose.

A solvent which has been effective in dissolution of cellulose and has a low vapor pressure similar to that of the ionic liquids is N-methyl morpholine N-oxide (NMMO). NMMO retains all the advantages of the ionic liquids ability to dissolve a variety of lignocellulosic substrates (Kuo and Lee 2009, Shafiei et al. 2010) without the need to chemically modify them and >99% of the solvent can be recovered due to its low vapor pressure (Perepelkin 2007). It is also nontoxic and biodegradable as proven by the work of Lenzing and other researchers (Rosenau et al. 2001). Further research is needed to evaluate and improve the economics of usage of ILs and NMMO for pretreatment of biomass. Pretreatment of lignocellulosic materials with acidified organic solvents (mixture of 80% ethylene glycol, 19.5% water and 0.5% HCl at 178°C for 90 min) has also been successfully used (Yamashita et al. 2010).The advantages of these methods include recovery and recycling of organic solvents as they can be easily distilled out. The disadvantages are that the process requires expensive high pressure equipment. Their performances could be improved by heating, microwave, or sonication (ElSeoud et al. 2007, Zhu et al. 2006a).

#### Physicochemical pretreatment methods

##### Steam-explosion

Steam-Explosion pretreatment is one of the most commonly used pretreatment options, as it uses both chemical and physical techniques in order to break the structure of the lignocellulosic material (McMillan 1994). This hydrothermal pretreatment method subjects the material to high pressures and temperatures for a short duration of time after which it rapidly depressurizes the system, disrupting the structure of cellulose microfibrils. The disruption of the fibrils increases the accessibility of the cellulose to the enzymes during hydrolysis.

Steam explosion is typically initiated at a temperature of 160–260°C (corresponding pressure 0.69–4.83 MPa) for several seconds to a few minutes before the material is exposed to atmospheric pressure. The process causes hemicellulose degradation and lignin transformation due to high temperature, thus increasing the potential of cellulose hydrolysis (Ballesteros et al. 2006, Chornet et al. 1988, Focher et al. 1991).

However, some disadvantages have been seen when using this process. Dilute acids are required to be added during softwood pretreatment or even when increased yields are warranted for lower acetylated feedstock. The factors that affect steam explosion pretreatment are residence time, temperature, chip size and moisture content (Duff and Murray 1996). Recent studies indicate that lower temperature and longer residence time are more favorable (Wright 1998).

#### Liquid hot water (LHW)

Much like the steam-explosion process, liquid hot water (LHW) pretreatment uses water at elevated temperatures and high pressures to maintain its liquid form in order to promote disintegration and separation of the lignocellulosic matrix. Temperatures can range from 160°C to 240°C over lengths of time ranging from a few minutes up to an hour with temperatures dominating the types of sugar formation and time dominating the amount of sugar formation (Yu et al. 2010).

This process has been found to be advantageous from a cost standpoint in that no additives such as acid catalysts are required. Furthermore, expensive reactor systems have not been necessary to use due to the low corrosive nature of this pretreatment technique. Neutralization of degradation products is not needed due to their fractionation and utilization in the liquid fraction. In the same sense, inhibitory products have not been reported to form overwhelmingly in the respective fractions allowing higher yields under specific conditions.

#### Ammonia fiber explosion (AFEX)

The ammonia fiber/freeze explosion (AFEX) process is another physicochemical process, much like steam explosion pretreatment, in which the biomass material is subjected to liquid anhydrous ammonia under high pressures and moderate temperatures and is then rapidly depressurized. The moderate temperatures (60°C to 100°C) are significantly less than that of the steam explosion process, thus allowing less energy input and overall cost reduction associated with the process (Alizadeh et al. 2005, Teymouri et al. 2004, Chundawat et al. 2007).

There have been extensive literature reviews on this type of pretreatment over the last decade, focusing on the advantages and disadvantages of the AFEX process used for different feedstocks (Sun and Cheng 2002, Mosier 2005). An overview of some of the advantages include lower moisture content, lower formation of sugar degradation products due to moderate conditions, 100% recovery of solid material, and the ability for ammonia to lessen lignin’s effect on enzymatic hydrolysis. A smaller number of disadvantages can be seen in the form of higher costs due to recycle and treatment of chemicals that are being used.

#### Ammonia recycle percolation (ARP)

Ammonia recycle percolation (ARP) has been paired with the AFEX pretreatment process by many authors, but it can have some different characteristics that need to be taken into consideration when looking at different pretreatment options (Kim and Lee 2005). In this process, aqueous ammonia of concentration between 5-15% (wt%) is sent through a packed bed reactor containing the biomass feedstock at a rate of about 5 ml/min. The advantage with this process over AFEX is its ability to remove a majority of the lignin (75–85%) and solubilize more than half of the hemicellulose (50–60%) while maintaining high cellulose content (Kim and Lee 2005). Primarily, herbaceous biomass has been most treated with this process: 60-80% delignification has been achieved for corn stover and 65–85% delignification for switchgrass (Iyer et al. 1996).

#### Supercritical fluid (SCF) pretreatment

A supercritical fluid is a material which can be either liquid or gas, used in a state above the critical temperature and critical pressure where gases and liquids can coexist. It shows unique properties that are different from those of either gases or liquids under standard conditions-it possesses a liquid like density and exhibits gas-like transport properties of diffusivity and viscosity (King and Srinivas 2009). Thus, SCF has the ability to penetrate the crystalline structure of lignocellulosic biomass overcoming the mass transfer limitations encountered in other pretreatments. The lower temperatures used in the process aids in the stability of the sugars and prevents degradation observed in other pretreatments. Kim and Hong 2001 investigated supercritical CO_2_ pretreatment of hardwood (Aspen) and southern yellow pine with varying moisture contents followed by enzymatic hydrolysis. SCF pretreatment showed significant enhancements in sugar yields when compared to thermal pretreatments without supercritical CO_2_. Alinia and coworkers ( 2010) investigated the effect of pretreatment of dry and wet wheat straw by supercritical CO_2_ alone and by a combination of CO_2_ and steam under different operating conditions (temperature and residence time in the reactors). It was found that a combination of supercritical CO_2_ and steam gave the best overall yield of sugars.

#### Biological pretreatment methods

Biological pretreatment uses microorganisms and their enzymes selectively for delignification of lignocellulosic residues and has the advantages of a low-energy demand, minimal waste production and a lack of environmental effects. In biological pretreatment processes, microorganisms such as brown-, white- and soft-rot fungi are used to degrade lignin and hemicellulose in waste materials Schurz ( 1978). White-rot basidiomycetes possess the capabilities to attack lignin. *Penicillium chrysosporium*, for example, has been shown to non-selectively attack lignin and carbohydrate (Anderson and Akin 2008). *P*. *chrysosporium* has been successfully used for biological pretreatment of cotton stalks by solid state cultivation (SSC) and results have shown that the fungus facilitates the conversion into ethanol (Shi et al., 2008). Brown rots mainly attack cellulose, while white and soft rots attack both cellulose and lignin. White-rot fungi are the most effective basidiomycetes for biological pretreatment of lignocellulosic materials (Fan et al. 1987). Other basidiomycetes such as *Phlebia radiata*, *P*. *floridensis* and *Daedalea flavida*, selectively degrade lignin in wheat straw and are good choices for delignification of lignocellulosic residues (Arora and Chander 2002). *Ceriporiopsis subvermispora*, however, lacks cellulases (cellobiohydrolase activity) but produces manganese peroxide and laccase, and selectively delignifies several different wood species (Ferraz 2003). The advantages of biological pretreatment include low energy requirement and mild environmental conditions. However, the rate of hydrolysis in most biological pretreatment processes is very low.

#### Hydrolysis of pretreated biomass

After pretreatment, the released cellulose and hemicelluloses are hydrolyzed to soluble monomeric sugars (hexoses and pentoses) using cellulases and hemicellulases, respectively. The initial conversion of biomass into sugars is a key bottleneck in the process of biofuel production and new biotechnological solutions are needed to improve their efficiency, which would lower the overall cost of bioethanol production. Enzymatic hydrolysis has been considered key to cost-effective bioethanol in the long run, and the reaction is carried out with mainly cellulase and hemicellulase for cellulose and hemicellulose, respectively. The advantages of using enzyme (cellulase) over acid is to eliminate corrosion problems and lower maintenance costs with mild processing conditions to give high yields.

Despite the fact that some fungal strains have the advantages of being thermostable and producing cellulases, most of these fungal strains do not produce sufficient amounts of one or more lignocellulolytic enzymes required for efficient bioconversion of lignocellulosic residues to fermentable sugars. In addition, plant cell walls are naturally resistant to microbial and enzymatic (fungal and bacterial) deconstruction, collectively known as ‘biomass recalcitrance’ (Himmel et al. 2007). These rate-limiting steps in the bioconversion of lignocellulosic residues to ethanol remain one of the most significant hurdles to producing economically feasible cellulosic ethanol. Improving fungal hydrolytic activity and finding stable enzymes capable of tolerating extreme conditions has become a priority in many recent studies.

#### Fungal extracellular cellulases

Enzymatic saccharification of lignocellulosic materials such as sugarcane bagasse, corncob, rice straw, *Prosopis juliflora*, *Lantana camara*, switch grass, saw dust, and forest residues by cellulases for biofuel production is perhaps the most popular application currently being investigated (Kuhad et al. 2010, Sukumaran et al. 2005). Both bacteria and fungi can produce glucanases (cellulases) that hydrolyze of lignocellulosic materials. These microorganisms can be aerobic or anaerobic and mesophilic or thermophilic. Bacteria belonging to genera of *Clostridium*, *Cellulomonas*, *Bacillus*, *Thermomonospora*, *Ruminococcus*, *Bacteriodes*, *Erwinia*, *Acetovibrio*, *Microbispora*, and *Streptomyces* are known to produce cellulase (Bisaria 1998). Anaerobic bacterial species such as *Clostridium phytofermentans*, *Clostridium thermocellum*, *Clostridium hungatei*, *and Clostridium papyrosolvens* produces cellulases with high specific activity (Duff and Murray 1996, Bisaria 1998). Most commercial glucanases (cellulases) are produced by *Trichoderma ressei* and β-D-glucosidase is produced from *Aspergillus niger* (Kaur et al. 2007). Fungi known to produce cellulases include *Sclerotium rolfsii*, *Phanerochaete chrysosporium* and various species of *Trichoderma*, *Aspergillus*, *Schizophyllum and Penicillium* (Sternberg 1976, Fan et al. 1987, Duff and Murray 1996). Among the fungi, *Trichoderma* species have been extensively studied for cellulase production (Sternberg 1976).

High temperature and low pH tolerant enzymes are preferred for the hydrolysis due to the fact that most current pretreatment strategies rely on acid and heat (Turner et al. 2007). In addition, thermostable enzymes have several advantages including higher specific activity and higher stability which improve the overall hydrolytic performance (Viikari et al. 2007). Ultimately, improvement in catalytic efficiencies of enzymes will reduce the cost of hydrolysis by enabling lower enzyme dosages. Some fungal strains such as *T*. *emersonii* (Grassick et al. 2004), *Chaetomium thermophilum* (Li et al. 2006) and *Corynascus thermophilus* (Rosgaard et al. 2006) can produce thermostable enzymes which are stable and active at elevated temperatures (60°C) well above their optimum growth temperature (30-55°C) (Maheshwari et al., 2000). Due to the promising thermostability and acidic tolerance of thermophilic fungal enzymes, they have good potential to be used for hydrolysis of lignocellulosic residues at industrial scales.

The anaerobic bacteria *Clostridium thermocellum* and *Clostridium cellulovorans* and the filamentous fungus *Trichoderma reesei* are well known as strongly cellulolytic and xylanolytic microorganisms. *C*. *thermocellum* and *C*. *cellulovorans* produce a cellulosome complex consisting of cellulase and hemicellulase organized on the cell surface (Demain et al. 2005); *T*. *reesei*, meanwhile, extracellularly secretes three types of cellulolytic enzyme, including five endoglucanases (EG [EC 3.2.1.4]) (Pere et al. 2001, Dienes et al. 2004), two cellobiohydrolases (CBH [EC 3.2.1.91]) (Bayer et al. 1998), and two β-glucosidases (BGL [EC 3.2.1.21]) (Sang-Mok and Koo 2001). Endoglucanases act randomly against the amorphous region of the cellulose chain to produce reducing and nonreducing ends for cellobiohydrolases, which produce cellobiose from reducing or nonreducing ends of crystalline cellulose. Cellulose chains are thus efficiently degraded to soluble cellobiose and cellooligosaccharides by the endo-exo synergism of EG and CBH (Hebeish and Ibrahim 2007). In the last step of enzymatic cellulose degradation, cellooligosaccharides are hydrolyzed to glucose by β-glucosidase. In addition to endo-exo synergism, exo-exo synergism between two cellobiohydrolases has also been reported.

#### Fungal hemicellulases

Several different enzymes are needed to hydrolyze hemicelluloses, due to their heterogeneity (Saha 2003). Xylan is the most abundant component of hemicellulose contributing over 70% of its structure. Xylanases are able to hydrolyze β-1,4 linkages in xylan and produce oligomers which can be further hydrolyzed into xylose by β-xylosidase. Not surprisingly, additional enzymes such as β-mannanases, arabinofuranosidases or α-L-arabinases are needed depending on the hemicellulose composition which can be mannan-based or arabinofuranosyl-containing. Also similarly to cellulases, most of the hemicellulases are glycoside hydrolases (GHs), although some hemicellulases belong to carbohydrate esterases (CEs) which hydrolyze ester linkages of acetate or ferulic acid side groups (Shallom and Shoham 2003). A mixture of hemicellulases or pectinases with cellulases exhibited a significant increase in the extent of cellulose conversion (Ghose and Bisaria 1979, Beldman et al. 1984). Many fungal species such as *Trichoderma*, *Penicillium*, *Aspergillus* and *T*. *emersonii* have been reported to produce large amounts of extracellular cellulases and hemicellulases.

#### Fungal ligninases

Fungi degrade lignin by secreting enzymes collectively termed “ligninases”. These include two ligninolytic families; i) phenol oxidase (laccase) and ii) peroxidases [lignin peroxidase (LiP) and manganese peroxidase (MnP)] (Martinez et al. 2005). White-rot basidiomycetes such as *Coriolus versicolor* (Wang et al. 2003), *P*. *chrysosporium* and *T*. *versicolor* (Moredo et al. 2003) have been found to be the most efficient lignin-degrading microorganisms studied. Interestingly, LiP is able to oxidize the non-phenolic part of lignin, but it was not detected in many lignin degrading fungi. In addition, it has been widely accepted that the oxidative ligninolytic enzymes are not able to penetrate the cell walls due to their size. Thus, it has been suggested that prior to the enzymatic attack, low-molecular weight diffusible reactive oxidative compounds have to initiate changes to the lignin structure and hemicellulose, fungal cellulosomes are much less well characterized compared to bacterial cellulosomes.

#### Fermentation

In the fermentation process, the hydrolytic products including monomeric hexoses (glucose, mannose and galactose) and pentoses (xylose and arabinose) will be fermented to valuable products such as ethanol. Among these hydrolytic products, glucose is normally the most abundant, followed by xylose or mannose and other lower concentration sugars.

The last two steps of bioconversion of pretreated lignocellulolytic residues to ethanol (hydrolysis and fermentation) can be performed separately (SHF) or simultaneously (SSF). In the separate hydrolysis and fermentation (SHF), the hydrolysate products will be fermented to ethanol in a separate process. The advantage of this method is that both processes can be optimized individually (e.g. optimal temperature is 45-50°C for hydrolysis, whereas it is 30°C for fermentation). However, its main drawback is the accumulation of enzyme-inhibiting end-products (cellobiose and glucose) during the hydrolysis. This makes the process inefficient, and the costly addition of β-glucosidase is needed to overcome end-product inhibition (Elumalia and Thangavelu 2010).

Further process integration can be achieved by a process known as consolidated bioprocessing (CBP) which aims to minimize all bioconversion steps into one step in a single reactor using one or more microorganisms. CBP operation featuring cellulase production, cellulose/hemicellulose hydrolysis and fermentation of 5- and 6- carbon sugars in one step have shown the potential to provide the lowest cost for biological conversion of cellulosic biomass to fuels, when processes relying on hydrolysis by enzymes and/or microorganisms are used (Lynd et al. 2005).

The simultaneous saccharification and fermentation (SSF) process was first studied by Takagi et al. ( 1977) for cellulose conversion to ethanol. The SSF process was originally developed for lignocellulosic biomass by researchers at Gulf Oil Company in 1974 (Blotkamo et al. 1978). The SSF process eliminates expensive equipment and reduces the probability of contamination by unwanted organisms that are less ethanol tolerant than the microbes selected for fermentation (Szczodrak 1989).

SSF combines the enzymatic saccharification of polymeric cellulose to simple monomeric forms such as glucose and its eventual fermentation by yeast to ethanol in the same vessel (Ikwebe and Harvey 2011). In simultaneous saccharification and fermentation (SSF), however, the end-products will be directly converted to ethanol by the microorganism. Therefore, addition of high amounts of β-glucosidase is not necessary and this reduces the ethanol production costs (Stenberg et al. 2000). Rapid conversion of the glucose into ethanol by yeast results in faster rates, higher yields, and greater ethanol concentrations than possible for SHF. The presence of ethanol in the fermentation broth also makes the mixture less vulnerable to invasion by unwanted microorganisms (Sasikumar and Viruthagiri 2010). However, the main drawback of SSF is the need to compromise processing conditions such that temperature and pH are suboptimal for each individual step. The development of recombinant yeast strains with improved thermotolerance can enhance the performance of SSF (Galbe and Zacchi 2002). It is reported that the major inefficiencies of biochemical process for lignocellulosic bioethanol production were identified as the simultaneous saccharification and fermentation (SSF) process accounting for 27% of the lost energy by thermodynamic analysis (Sohel and Jack 2010). Alkasrawi et al. ( 2003) reported that addition of surfactants as an additive in SSF can significantly lower the operational cost of the process because it increases the conversion rate of cellulose to glucose. Addition of Tween-20, 2.5 g/l not only reduces the time required to attain maximum ethanol concentration, but also enhances enzyme activity in the liquid fraction at the end of SSF, probably by preventing unproductive binding of the cellulases to lignin, which could facilitate enzyme recovery.

Over the years, various groups have worked on the SSF process to improve the choice of enzymes, fermentative microbes, biomass pretreatment, and process conditions. Extensive studies on SSF have since been conducted focusing on the production of ethanol from cellulosic substrates. Phillipidis et al. ( 1993) have studied the enzymic hydrolysis of cellulose in an attempt to optimize SSF performance. Ghose et al. ( 1984) have increased ethanol productivity by employing a vacuum cycling in an SSF process using lignocellulosic substances. Zhu et al. ( 2006b) evaluated the suitability of production of ethanol from the microwave-assisted alkali pretreated wheat straw, the simultaneous saccharification and fermentation (SSF) of the microwave-assisted and conventional alkali pretreated wheat straw to ethanol.

*Candida brassicae* is accepted as the yeast of choice as far as SSF is considered, although both *Saccharomyces cerevisiae* and *S*. *carlsbergensis* have been found to offer similar rates. Several other yeasts as well as the bacteria *Zymomonas mobilis* have been studied with cellulose from *T*. *ressei* mutants for SSF processes. Researchers have also examined several combinations of enzymes with *Z*. *mobilis*, *S*. *cerevisiae*, and other ethanol producer, but they have only considered substrate levels lower than necessary to prove economic viability. Wyman et al. ( 1986) evaluated the cellobiose-fermenting yeast *Brettanomyces clausenii* for the SSF of cellulose to ethanol.

There are number of different methods to quantitate ethanol in samples. HPLC has been utilized to monitor the fermentation process This method has the advantage of being able to monitor not only the production of ethanol, but also the reaction substrates and byproducts (Hall and Reuter 2007). Fourier transform infrared spectroscopy (Sharma et al. 2009), gas chromatography (Wang et al. 2003), and Infrared (Lachenmeier et al. 2010) technologies have also been used to detect and quantitate ethanol in samples. While FTIR requires a large investment in instrumentation, the use or less expensive IR technology has been demonstrated to be just as accurate (Lachenmeier et al. 2010). Gerchman et al. ( 2012) developed a cheap and rapid approach for ethanol quantification in aqueous media during fermentation steps as part of the conversion of biomass to ethanol. The suggested method requires a sample of a small volume and consists of organic extraction, followed by direct use of gas chromatography with a flame ionization detector (GC-FID). The feasibility of such approach is obvious since there is no need for the head-space system, distillation, expensive reagents and sophisticated equipment. The proposed method was also tested for its ‘real-life’ applicability for ethanol quantification from fermentation process.

According to Keller and Bryan ( 2000), distillation is still a “formidable competitor” as a major separation method even though much research has been thrust on its alternatives. Hence, distillation, especially simple distillation, tends to be the first choice in industry for separating a liquid mixture; other methods, including complex distillation, e.g., azeotropic distillation, come into play only when simple distillation is deemed to be technically infeasible or economical inviable because of typically three large stainless steel distillation towers, stainless steel heat exchangers and price of stainless up 400% in last six years, high operating costs because 280 MMBTU/hr energy is consumed (100 MGPY ethanol). Mole sieve drying adds to energy costs and that’s why energy costs up significantly with price of crude oil.

Under certain circumstances, retrofitting of an existing process can be economically far more viable than constructing a new process, especially when the financial resources are limited and/or when short term needs are to be met under a tight time constraint. Developing economically viable fermentation processes requires efficient downstream processing: selective product removal and avoiding byproduct streams. “ESepis a modular, low-energy process for the recovery of ethanol from fermentation broth with an estimated reduction of up to 60% in both capital and operating costs versus conventional distillation. Use of non-stainless steel components also results in a substantial reduction in construction time”. It is applicable to new ethanol plants (corn, sugar and cellulosic). It replaces whole distillation train and mole sieve dryer. With new plants it reduces overall energy consumption by >60% (ESep 2008).

The utilization of pervaporation for the production of absolute (anhydrous) ethanol through its coupling with the previous distillation step has been reported. The modeling and optimization of the process using MINLP tools showed 12% savings in the production costs considering a 32% increase in membrane area and the reduction in both reflux ratio and ethanol concentration in the distillate of the column (Lelkes et al. 2000, Szitkai et al. 2002). Through pilot-plant studies, the integration of distillation process with the pervaporation has been achieved resulting in good indexes in terms of energy savings. These savings are due to the low operation costs of pervaporation and to the high yield of dehydrated ethanol, typical of pervaporation processes (Tsuyomoto et al. 1997). The comparison between azeotropic distillation using benzene and pervaporation system using multiple membrane modules showed that, at the same ethanol production rate and quality (99.8 wt.%), operation costs, including the membrane replacement every 2–4 years, are approximately 1/3–1/4 of those of azeotropic distillation.

#### Methods used to improve fungal enzyme production, activity and/or stability

In order to produce ethanol industrially, the fermentative microorganism needs to be robust. The utilization of all the sugars generated from lignocellulosic hydrolysate is essential for the economical production of ethanol (Saha 2003). The conventional ethanol fermenting yeast (*Saccharomyces cerevisiae*) or bacterium (*Zymomonas mobilis*) cannot ferment multiple sugar substrates to ethanol (Bothast et al. 1999). A major technical hurdle to converting lignocellulose to ethanol is developing an appropriate microorganism for the fermentation of a mixture of sugars such as glucose, xylose, arabinose, and galactose (Bothast et al. 1999). A number of recombinant microorganisms such as *Escherichia coli*, *Klebsiella oxytoca*, *Z*. *mobilis*, and *S*. *cerevisiae* have been developed over the last 25 years with a goal of fermenting mixed sugars to ethanol (Alterthum and Ingram 1989, Ohta et al. 1991, Zhang et al. 1995, Ho et al. 1998). Saha and Cotta’s ( 2011) research unit has developed a recombinant *E*. *coli* (strain FBR5) that can ferment mixed multiple sugars to ethanol (Dien et al. 2000). The strain carries the plasmid pLOI297, which contains the genes for pyruvate decarboxylase (pdc) and alcohol dehydrogenase (adh) from *Z*. *mobilis* necessary for efficiently converting pyruvate into ethanol ( Alterthum and Ingram 1989).

Technologies required for bioconversion of lignocelluloses to ethanol and other valuable products are currently available but need to be developed further in order to make biofuels cost competitive compared to other available energy resources such as fossil fuels. The most recent and important improvements in production/activity of fungal enzymes using different techniques such as mutagenesis, co-culturing and heterologous gene expression of cellulases are discussed below.

#### Mutagenesis

Many fungal strains have been subjected to extensive mutagenesis studies due to their ability to secrete large amounts of cellulose-degrading enzymes. Cellulolytic activity of *T*. *reesei* QM6a has been improved by using different mutagenesis techniques including UV-light and chemicals, resulting in the mutant QM 9414 with higher filter paper activity (FPA) (Mandels et al. 1971). *T*. *reesei* RUT-C30 is one of the best known mutants, producing 4–5 times more cellulase than the wild-type strain (QM 6a). A recent study by Kovacs et al. 2008 has shown that wild-type *Trichoderma atroviride* (F-1505) produces the most cellulase among 150 wild-type *Trichoderma*. Moreover, *T*. *atroviride* mutants were created by mutagenesis using *N*-methyl-*N*’-nitro-*N*-nitrosoguanidine (NTG) as well as UV-light. These *T*. *atroviride* mutants (e.g. *T*. *atroviride* TUB F-1724) produce high levels of extracellular cellulases as well as β-glucosidase when they are grown on pretreated willow. Cellulase and xylanase activities in *Penicillium verruculosum* 28 K mutants were improved about 3-fold using four cycles of UV mutagenesis. The enzyme production was further improved by 2- to 3-fold in a two-stage fermentation process using wheat bran, yeast extract medium and microcrystalline cellulose as the inducer (Soloveva et al. 2005).

Site-directed mutagenesis (SDM) plays a central role in the characterization and improvement of cellulases including their putative catalytic and binding residues. The application of SDM it was found that Glu 116 and 200 are the catalytic nucleophile and acid–base residues in *Hypocrea jecorina* (anamorph *T*. *reesei*) Cel12A, respectively. In the study, mutant enzymes were produced where Glu was replaced by Asp or Gln at each position (E116D/Q and E200D/Q). The specific activity of these mutants was reduced by more than 98%, suggesting the critical role of these two residues in the catalytic function of the enzyme (Okada et al. 2000). In another study, the thermostable endo-1,4-β-xylanase (XynII) mutants from *T*. *reesei* were further mutated to resist inactivation at high pH by using SDM. All mutants were resistant to thermal inactivation at alkaline pH. For example, thermotolerance for one mutant (P9) at pH 9 was increased approximately 4–5°C, resulting in better activity in sulphate pulp bleaching compared to the reference (Fenel et al. 2006). Also, the catalytic efficiency and optimum pH of *T*. *reesei* endo-β-1,4-glucanase II were improved by saturation mutagenesis followed by random mutagenesis and two rounds of DNA shuffling. The pH optimum of the variant (Q139R/L218H/W276R/N342T) was shifted from 4.8 to 6.2, while the enzyme activity was improved more than 4.5-fold (Qin et al. 2008). Moreover, the stability of *T*. *reesei* endo-1,4-β-xylanases II (XynII) was increased by engineering a disulfide bridge at its N-terminal region. In fact, two amino acids (Thr-2 and Thr-28) in the enzyme were substituted by cysteine (T2C:T28C mutant) resulting in a 15°C increase in thermostability (Fenel et al. 2004).

#### Co-culturing

Fungal co-culturing offers a means to improve hydrolysis of lignocellulosic residues, and also enhances product utilization which minimizes the need for additional enzymes in the bioconversion process. In the case of cellulose degradation, for example, all three enzymatic components (EG, CBH and β-glucosidase) have to be present in large amounts. However, none of the fungal strains, including the best mutants, are able to produce high levels of the enzymes at the same time. *T*. *reesei* for example produces CBH and EG in high quantities whereas its β-glucosidase activity is low (Stockton et al. 1991). *A*. *niger* however, produces large amounts of β-glucosidase, but has limited EG components (Kumar et al. 2008). In addition, hemicellulose hydrolysis must also be considered when lignocellulosic residues are subjected to biomass conversion. However, this will be determined by the pretreatment methods. Specifically in an alkali pretreatment method, a part of lignin will be removed and thus hemicellulose has to be degraded by the use of hemicellulases, whereas in acid-catalyzed pretreatment, the hemicellulose layer will be hydrolyzed (Hahn et al. 2006). Again, some fungal strains have been shown to work more efficiently on cellulosic residues whereas others produce more hemicellulolytic enzymes and efficiently hydrolyze hemicellulosic portions (Howard et al. 2003). Conversion of both cellulosic and hemicellulosic hydrolytic products in a single process can be achieved by co-culturing two or more compatible microorganisms with the ability to utilize the materials. In fact, in nature, lignocellulosic residues are degraded by multiple co-existing lignocellulolytic microorganisms.

Mixed fungal cultures have many advantages compared to their monocultures, including improving productivity, adaptability and substrate utilization. Improving fungal cellulolytic activity of *T*. *reesei* and *A*. *niger* by co-culturing was the subject of extensive research including studies done by Maheshwari et al. ( 1994), Ahamed et al. ( 2008) and Juhasz et al. 2003. Moreover, other fungal strains have been co-cultured to obtain better cellulolytic activity such as co-culturing of *T*. *reesei* RUT-C30 and *A*. *phoenicis* (Duff 1985) or *A*. *ellipticus* and *A*. *fumigatus* (Gupte and Madamwar 1997). There are a few examples of co-culturing fungal strains for the purpose of combining cellulose and hemicellulose hydrolysis such as co-culturing *T*. *reesei* D1-6 and *A*. *wentii* Pt 2804 in a mixed submerged culture (Panda et al. 1983) or co-culturing *T*. *reesei* LM-UC4 and *A*. *phoenicis* QM329 using ammonia-treated bagasse (Duenas et al. 1995). In the both cases, enzyme activity for cellulases and hemicellulases was significantly increased. The main drawback of co-culturing however is the complexity of growing multiple microorganisms in the same culture (Lynd et al. 2002).

#### Metabolic engineering

Metabolic engineering is a powerful method to improve, redirect, or generate new metabolic reactions or whole pathways in microorganisms. This enables one microorganism to complete an entire task from beginning to end. This can be done by altering metabolic flux by blocking undesirable pathway(s) and/or enhancement of desirable pathway(s). For example by application of homologous recombination, the production of *T*. *reesei* β-glucosidase I was enhanced using xylanase (*xyn3*) and cellulase (*egl3*) promoters which improved β-glucosidase activity to 4.0 and 7.5 fold compared to the parent, respectively. This will permit one fungal strain such as *T*. *reesei* to be more efficient on hydrolysis of cellulose to glucose which improve the yield and therefore lower the cost (Rahman et al. 2009). Becker and Boles ( 2003) described the engineering of a *Saccharomyces cerevisiae* strain able to utilize the pentose sugar l-arabinose for growth and to ferment it to ethanol. Expanding the substrate fermentation range of *S*. *cerevisiae* to include pentoses is important for the utilization of this yeast in economically feasible biomass-to-ethanol fermentation processes. After overexpression of a bacterial l-arabinose utilization pathway consisting of *Bacillus subtilis* AraA and *Escherichia coli* AraB and AraD and simultaneous overexpression of the l-arabinose-transporting yeast galactose permease, we were able to select an l-arabinose-utilizing yeast strain by sequential transfer in l-arabinose media. High l-arabinose uptake rates and enhanced transaldolase activities favor utilization of l-arabinose.

Shaw et al. 2008 engineered *Thermoanaerobacterium saccharolyticum*, a thermophilic anaerobic bacterium that ferments xylan and biomass-derived sugars, to produce ethanol at high yield. Knockout of genes involved in organic acid formation (acetate kinase, phosphate acetyltransferase, and L-lactate dehydrogenase) resulted in a strain able to produce ethanol as the only detectable organic product and substantial changes in electron flow relative to the wild type. Glucose and xylose are co-utilized and utilization of mannose and arabinose commences before glucose and xylose are exhausted.

#### Heterologous expression

Heterologous expression is a powerful technique to improve production yield of enzymes, as well as activity. In order to make a robust lignocellulolytic fungal strain, many different fungal cellulases with higher and/or specific activity based on the need for a functional cellulase system in the organism have been cloned and expressed. For example, thermostable β-glucosidase (*cel3a*) from thermophilic fungus *T*. *emersonii* was expressed in *T*. *reesei* RUT-C30 using a strong *T*. *reesei cbh1* promoter. The expressed enzyme has been shown to be highly thermostable (optimum temperature at 71.5°C) with high specific activity (Murray et al. 2004). In the study for the improvement of biofinishing of cotton, *T*. *reesei* cellobiohydrolase (I & II) were overexpressed using additional copy(s) of the genes cloned under *T*. *reesei cbh1* promoter. The results have shown that the expression of CBHI was increased to 1.3- and 1.5-fold with one or two additional copies of the gene, respectively.

#### Immobilization

Immobilization of microbial cells and enzymes have showed certain technical and economical advantages over free cell system. Using immobilized enzymes not only leads to greater product purity, cleaner processes, and economic operational costs but also makes the use enzyme cost effective and recoverable (Meena and Raja 2004). The immobilized biocatalysts have been extensively investigated during last few decades. An immobilized cellobiase enzyme system has been used in the enzymatic hydrolysis of biomass for the generation of cellulosic ethanol (Das et al., 2011). Production of alcohol and biodiesel fuel from triglycerides using immobilized lipase has been carried out using porous kaolinite particle as a carrier (Iso et al. 2001).

The use of an immobilized yeast cell system for alcoholic fermentation is an attractive and rapidly expanding research area because of its additional technical and economical advantages compared with the free cell system. A reduction in the ethanol concentration in the immediate microenvironment of the organism due to the formation of a protective layer or specific adsorption of ethanol by the support may act to minimize end product inhibition. The most significant advantages of immobilized yeast cell systems are the ability to operate with high productivity at dilution rates exceeding the maximum specific growth rate, the increase of ethanol yield and cellular stability and the decrease of process expenses due to the cell recovery and reutilization (Lin and Tanaka 2006). Other advantages of immobilized cell system over presently accepted batch or continuous fermentations with free-cells are: greater volumetric productivity as a result of higher cell density; tolerance to higher concentrations of substrate and products; lacking of inhibition; relative easiness of downstream processing etc. in different types of bioreactors, such as packed bed reactor, fluidized bed reactor, gaslift reactor and reactor with magnetic field (Ivanova et al. 1996, Sakai et al. 1994; Perez et al. 2007).

Perspective techniques for yeasts immobilization can be divided into four categories: attachment or adsorption to solid surfaces (wood chips, delignified brewer’s spent grains, DEAE cellulose, and porous glass), entrapment within a porous matrix (calcium alginate, k-carrageenan, polyvinyl alcohol, agar, gelatine, chitosan, and polyacrilamide), mechanical retention behind a barrier (microporous membrane filters, and microcapsules) and self-aggregation of the cells by flocculation (Ivanova et al. 2011).

#### Process integration

One of the most important approaches for the design of more intensive and cost-effective process configurations is process integration. Process integration looks for the integration of all operations involved in the production of fuel ethanol. This can be achieved through the development of integrated bioprocesses that combine different steps into one single unit. Thus, reaction–separation integration by removing ethanol from the zone where the biotransformation takes place, offers several opportunities for increasing product yield and consequently reducing product costs. Other forms of integration may significantly decrease energetic costs of specific flowsheet configurations for ethanol production. Process integration is gaining more and more interest due to the advantages related to its application in the case of ethanol production: reduction of energy costs, decrease in the size and number of process units, intensification of the biological and downstream processes. Integration of fermentation and separation processes for reduction of product inhibition, development of efficient cogeneration technologies using cane bagasse, development of CBP, application of membrane technology (e.g. for ethanol removal or dehydration) are examples of process integration.

## Conclusion

Lignocellulolytic microorganisms, especially fungi, have attracted a great deal of interest as biomass degraders for large-scale applications due to their ability to produce large amounts of extracellular lignocellulolytic enzymes. Many successful attempts have been made to improve fungal lignocellulolytic activity including recombinant and non-recombinant techniques. Process integration has also been considered for the purpose of decreasing the production cost, which was partly achieved by performing hydrolysis and fermentation in a single reactor (SSF) using one or more microorganisms (co-culturing).

These laboratory improvements should now be verified in pilot and demonstration plants. Scaling up the production of lignocellulosic ethanol, however, requires further reduction of the production cost. Thus, in order to improve the technology and reduce the production cost, two major issues have to be addressed: i) improving technologies to overcome the recalcitrance of cellulosic biomass conversion (pretreatment, hydrolysis and fermentation) and ii) sustainable production of biomass in very large amounts.

### Future prospects

It is considered that lignocellulosic waste will become the main feedstock for ethanol production in the near future. In the case of large scale biomass production, additional waste stocks can be tested and used as substrates to meet the needs. On the other hand, biotechnological approaches including systems biology and computational tools are likely good candidates to overcome these issues. Future trends for costs reduction should include more efficient pretreatment of biomass, improvement of specific activity and productivity of cellulases, improvement of recombinant microorganisms for a greater assimilation of all the sugars released during the pretreatment and hydrolysis processes, and further development of co-generation system. Undoubtedly, ongoing research on genetic and metabolic engineering will make possible the development of effective and stable strains of microorganisms for converting cellulosic biomass into ethanol. Process engineering will play a central role for the generation, design, analysis and implementation of technologies improving the indexes of global process, or for the retrofitting of employed bioprocesses. Undoubtedly, process intensification through integration of different phenomena and unit operations as well as the implementation of consolidated bioprocessing of different feedstocks into ethanol (that requires the development of tailored recombinant microorganisms), will offer the most significant outcomes during the search of the efficiency in fuel ethanol production. This fact will surely imply a qualitative improvement in the industrial production of fuel ethanol in the future.

## Competing interests

The authors declare that they have no competing interests.
